# Feasibility of video-based real-time nystagmus tracking: a lightweight deep learning model approach using ocular object segmentation

**DOI:** 10.3389/fneur.2024.1342108

**Published:** 2024-02-21

**Authors:** Changje Cho, Sejik Park, Sunmi Ma, Hyo-Jeong Lee, Eun-Cheon Lim, Sung Kwang Hong

**Affiliations:** ^1^Hallym University Medical Center, DIDIM Research Institute, Chuncheon, Republic of Korea; ^2^Department of Otorhinolaryngology-Head and Neck Surgery, Hallym University College of Medicine, Chuncheon, Republic of Korea

**Keywords:** vertigo, artificial intelligence (AI), CDSS, nystagmus, feasibilities studies, segmentation, pupil center, blink

## Abstract

**Background:**

Eye movement tests remain significantly underutilized in emergency departments and primary healthcare units, despite their superior diagnostic sensitivity compared to neuroimaging modalities for the differential diagnosis of acute vertigo. This underutilization may be attributed to a potential lack of awareness regarding these tests and the absence of appropriate tools for detecting nystagmus. This study aimed to develop a nystagmus measurement algorithm using a lightweight deep-learning model that recognizes the ocular regions.

**Method:**

The deep learning model was used to segment the eye regions, detect blinking, and determine the pupil center. The model was trained using images extracted from video clips of a clinical battery of eye movement tests and synthesized images reproducing real eye movement scenarios using virtual reality. Each eye image was annotated with segmentation masks of the sclera, iris, and pupil, with gaze vectors of the pupil center for eye tracking. We conducted a comprehensive evaluation of model performance and its execution speeds in comparison to various alternative models using metrics that are suitable for the tasks.

**Results:**

The mean Intersection over Union values of the segmentation model ranged from 0.90 to 0.97 for different classes (sclera, iris, and pupil) across types of images (synthetic vs. real-world images). Additionally, the mean absolute error for eye tracking was 0.595 for real-world data and the F1 score for blink detection was ≥ 0.95, which indicates our model is performing at a very high level of accuracy. Execution speed was also the most rapid for ocular object segmentation under the same hardware condition as compared to alternative models. The prediction for horizontal and vertical nystagmus in real eye movement video revealed high accuracy with a strong correlation between the observed and predicted values (*r* = 0.9949 for horizontal and *r* = 0.9950 for vertical; both *p* < 0.05).

**Conclusion:**

The potential of our model, which can automatically segment ocular regions and track nystagmus in real time from eye movement videos, holds significant promise for emergency settings or remote intervention within the field of neurotology.

## Introduction

Sudden damage to the peripheral-to-central vestibular pathway can lead to visual fixation failure due to the loss of the vestibulo-ocular reflex, resulting in abnormal eye movements accompanied by acute vertigo. The observation of such movements provides diagnostic information regarding the underlying lesion. For instance, physicians can identify the neural substrate responsible for acute vertigo using the “HINTS” examination, i.e., the head impulse (HI) and nystagmus tests (N), and the test of skew (TS). These tests are widely used to identify central lesions in patients with acute vestibular syndrome (1–3). Recent studies have revealed that the “HINTS” examination is more sensitive than neuroimaging modalities for distinguishing patients with central lesions such as posterior circulation strokes among patients with acute vertigo; the sensitivity is 100% and the specificity is 90–94.4% within the first 24 h after a vertigo attack (1, 4). Additionally, nystagmus evaluation is critical when diagnosing benign paroxysmal positional vertigo (BPPV) or inferring the inner ear status in patients with Meniere's disease. Thus, neuro-otologists must aim to identify abnormal eye movements as the first bedside exam in patients with acute vertigo.

However, performing eye movement analyses, including HINTS, and specifically interpreting them, require training to achieve the clinical accuracy necessary for making diagnoses. Unfortunately, most patients with acute vertigo primarily visit emergency departments (EDs) or primary health care units (PHCUs) that lack trained experts to diagnose with confidence (5, 6). Furthermore, observation of eye movements during the ictal period is critical but difficult if no neurotologist or device to track eye movements is available at the time of the vertigo attack. Therefore, a focus on providing a dedicated support system in eye movement tracking and interpretation to frontline physicians could contribute to improving diagnostic workflow and avoiding unnecessary brain imaging. We hypothesized that recording eye movements by patients themselves during episodes of vertigo and subsequently presenting the video footage along with nystagmus tracking results during doctor's appointments could significantly improve healthcare access and patient care. This approach would be particularly valuable for remote evaluation in rural areas where specialized medical expertise may be limited.

We previously developed a deep learning-based diagnostic system that classified nystagmus patterns in BPPV patients, in which we speculated that our trained model from those who revealed horizontal, vertical, or torsional nystagmus according to the affected canal can support frontline physicians in making a diagnosis for patients with vertigo (7). However, we have reached the conclusion that any model that seeks to address these problems must be lightweight and patient-centric design to facilitate efficient diagnostic workflow. Recent studies have utilized webcams (8) and smartphones (9) as well as video-oculographic devices (10) for detection of nystagmus. Thus, we have developed a new technology capable of continuously tracking eye movements by recognizing regions of the ocular. It is also capable of providing frame-by-frame inferences even on low-end graphics processing units (GPUs) without sacrificing horizontal or vertical nystagmus tracking accuracy.

We hope that our model can expedite the diagnostic process for patients with vertigo, particularly in EDs where timely decisions are critical or PHCUs, and contribute to remote consultations with neurotologic experts, bridging geographic variations and ensuring interpretation of eye movements without the need for additional specialized equipment, for patients in rural or underserved areas.

## Materials and methods

### Datasets and preprocessing

We obtained extracted images from real eye movement video clips from Hallym University Sacred Heart Hospital (HUSHH) and real eye-image datasets from OpenEDS (11) while synthetic eye-image datasets were obtained from UnityEyes (12) (Table 1). The HUSHH datasets were eye-tracking videos of patients (*N* = 1,568) who underwent the spontaneous nystagmus test and video head impulse test (Interacoustics, Middelfart, Denmark); the Institutional Review Board of Hallym University College of Medicine approved our use of the datasets (#2022-02-012).

**Table 1:** Image datasets for model development.

**Source**	**Training**	**Validation**	**Test**	**Total**
HUSHH-SN	686	86	86	858
HUSHH-vHIT	566	71	71	708
OpenEDS	9,311	1,164	1,164	11,639
UnityEyes	55,267	6,909	6,908	69,084
Total	65,830	8,229	8,230	82,289

SN, spontaneous nystagmus; vHIT, video head impulse test; HUSHH, Hallym University Sacred Heart Hospital.

During the data generation process for extraction of images within the videos from HUSHH, we assessed image similarity using the Structural Similarity Index Measure (SSIM) from the Scikit-image package (13). Images with low similarity (SSIM below 0.8) were selectively extracted, ensuring the exclusion of similar instances throughout the entire dataset. OpenEDS data were collected during challenges conducted by Meta Platforms Inc. (CA, USA); the Sparse Semantic Segmentation Challenge 2020 datasets were used with permission. Synthetic eye images using UnityEyes were generated with the starting position of the pupil center set at (x, y) = (0, 0), and the maximum allowed variations were configured to span from −40 to +40° for both the x and y axes. These settings were utilized to replicate various real eye movements based on data from UnityEyes.

**Figure 1:**
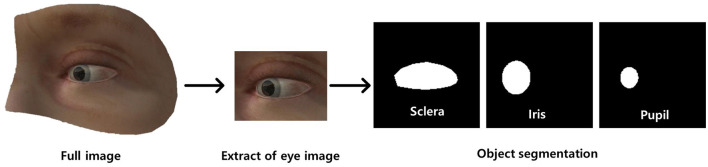
Dataset preprocessing (annotation) for ocular object segmentation model. There were two preprocessing steps: (a) extraction of the ocular region from the image followed by (b) labeling of the sclera, iris, and pupil.

We used a total of 82,289 images from those datasets for eye tracking and segmentation of ocular regions, which was split into 80% for the training set, 10% for the validation set, and 10% for the testing set according to their data sources (Table 1 and Figure 1). All images were resized to 100 × 100 pixels.

Subsequently, we manually annotated ocular regions on those images using segmentation masks of the sclera, iris, and pupil (Figure 1). In addition, gaze vectors were employed for eye tracking, with (x, y) coordinates of the pupil center serving as the basis for tracking the direction of gaze. The gold standard for eye landmark annotation, specifically for the iris and pupil, involves fitting an ellipse to the segmentation area. The center point is determined by calculating the centroid of the fitted ellipse, and this derived center point is utilized as the labeled position. This approach offers objectivity and precision compared to manual labeling of the center point by individuals.

For the blink detection model, a total of 23,954 images out of 82,289 images were annotated as follows; “open” when the iris was fully visible, “closing” when the iris was partially covered by the eyelid, and “closed” when the iris was invisible. The images are split into 80% for the training set, 10% for the validation set, and 10% for the testing set (Table 2).

**Table 2:** Datasets for blink classification.

**Category**	**Training**	**Validation**	**Test**	**Total**
Open	6,697	837	838	8,372
Closing	7,045	881	881	8,807
Closed	5,420	677	678	6,775
Total	19,162	2,395	2,397	23,954

### Model architecture

**Figure 2:**
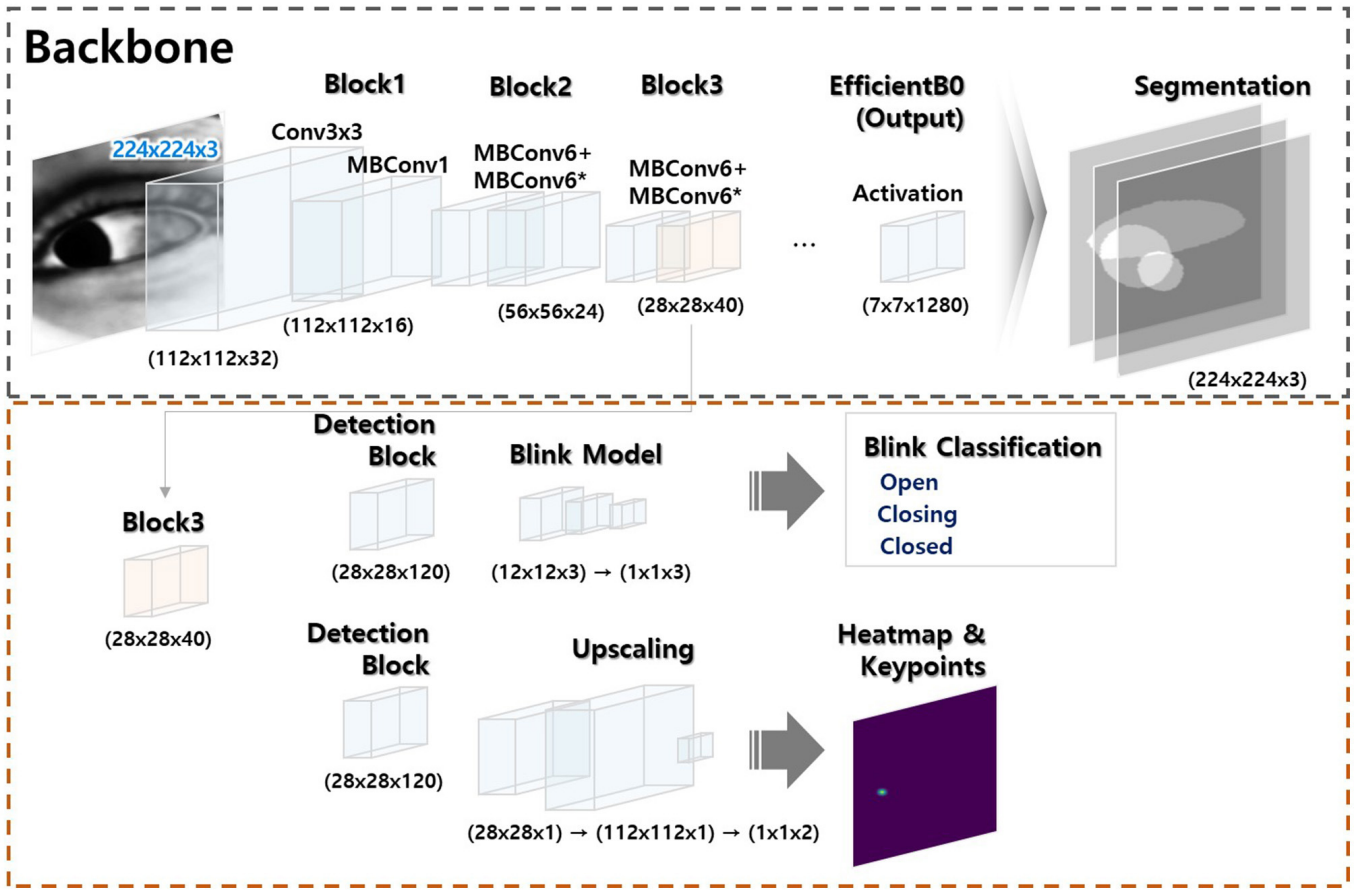
Model architecture. The model has two modules: (a) a segmentation module (black dotted square) and (b) a module that classifies key object points (red dotted square). The former module is trained to allow artificial intelligence-based generation of key ocular variables. The latter module uses a portion of the former module to combine or separate the segmentation outputs; integration is flexible (the Supplementary material provides a comprehensive overview of the methods employed for ocular object segmentation, blink detection, and eye-tracking).

Initially, our deep learning models were developed independently according to their specific tasks (a model that segmented the sclera, iris, and pupil, a gaze estimation model, and a model that detected blinks; Please refer to the Supplementary material for more detailed information regarding three model developments). Subsequently, these models were integrated for the nystagmus tracking (Figure 2).

**Figure 3:**
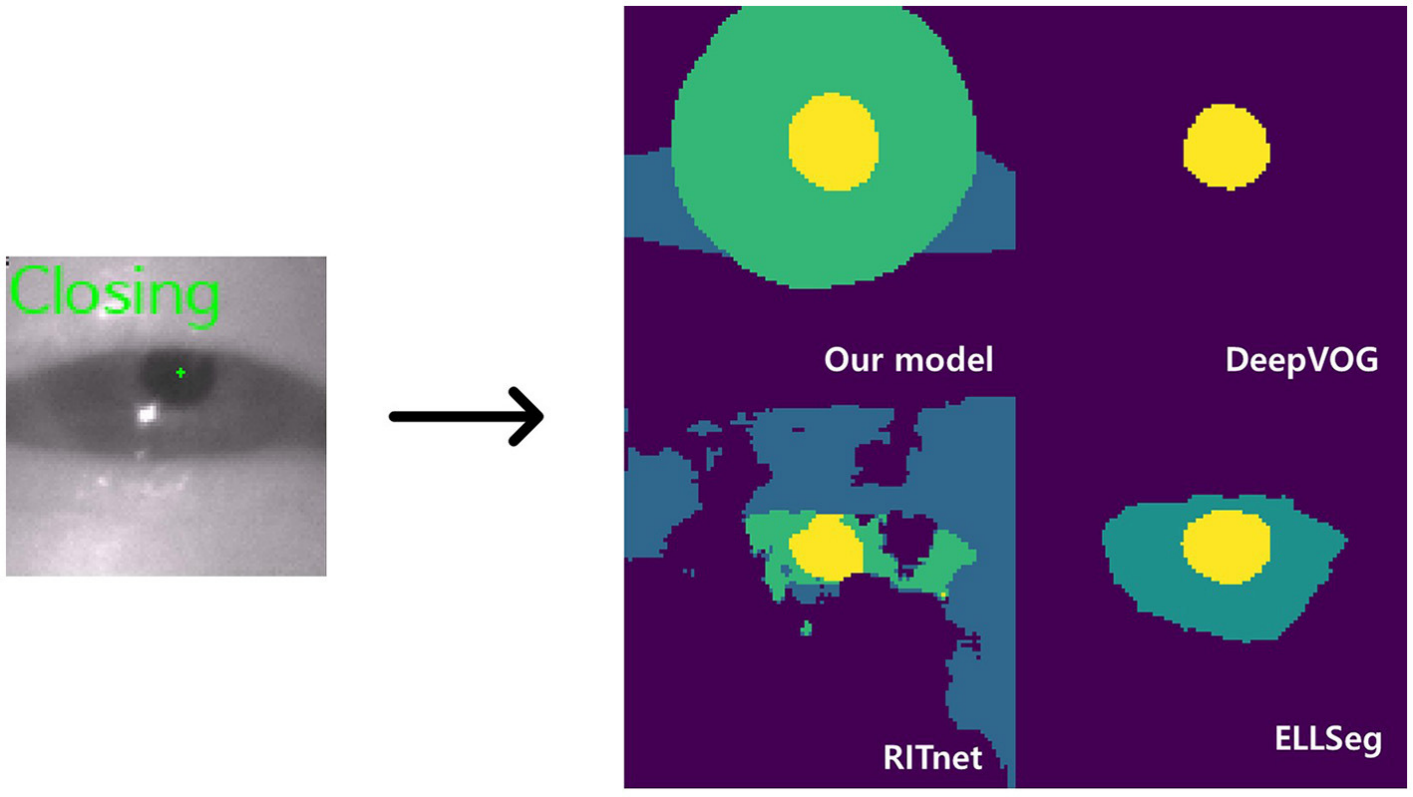
Model inference. The left image shows a closing blink and the pupil center points detected by our model. The images on the right are the AI outputs of the sclera, iris, and pupil (DeepVOG lacks iris and scleral outputs, and EllSeg has no scleral output).

**Figure 4:**
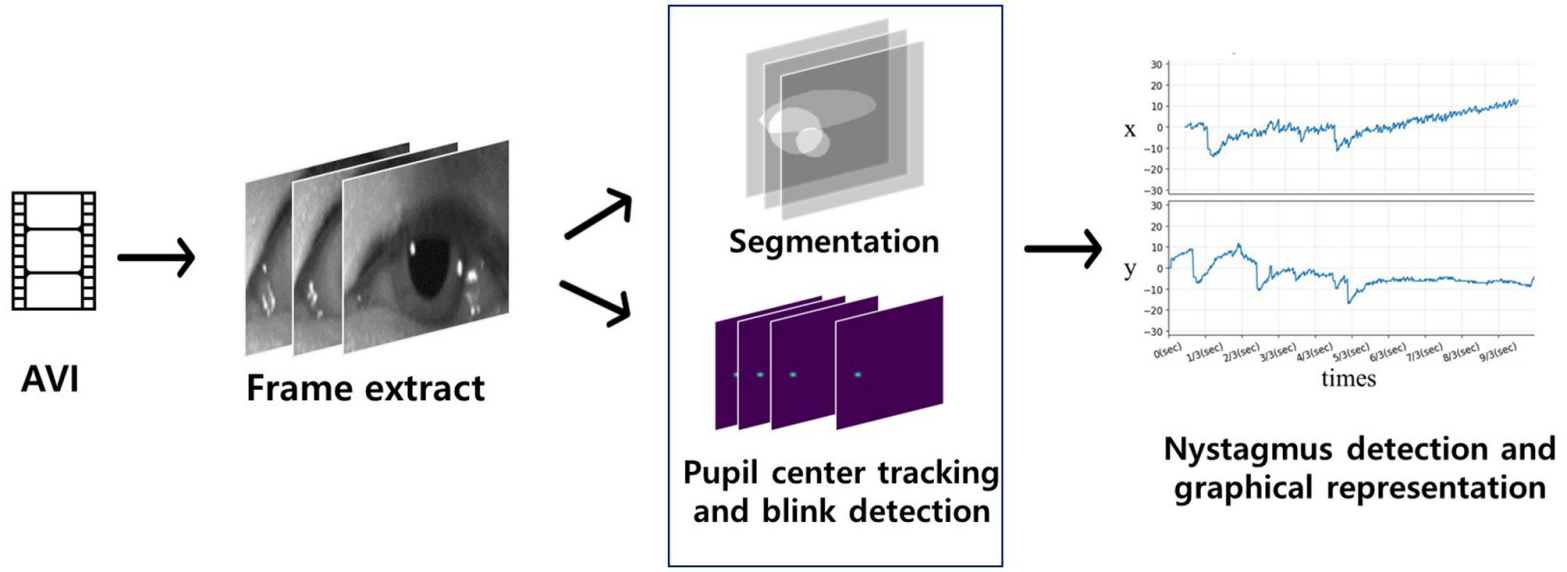
Flow of the deep learning-based nystagmus-tracking algorithm. Video frames of eye movements were input for segmentation and detection of the pupil center and blinking (blue quadrangle). Significant eye movements (nystagmus) were plotted on the x (horizontal eye movements) and y axes (vertical eye movements).

The algorithm flow of deep learning-based eye movement analysis can be summarized as follows: (1) input video data; (2) extract frames; (3) input the frames; (4) output the segmented pupil, iris, eyelid, blink status (open, closing, closed), and (x, y) coordinates of the iris center (Figure 3); and (5) graphically present nystagmus patterns on the horizontal and vertical axes (Figure 4).

In more detail, the algorithm started by locating the pupil centers in eye movement videos. Frame images were extracted from these videos, serving as input data for artificial intelligence (AI) to identify the central pupil coordinates and detect whether the eyes were blinking. For each frame where the eye was not closed, horizontal (x) and vertical (y) coordinates were extracted, referencing the central pupil coordinates, and plotted over time. In cases where the eye was closed, the algorithm interpolated the horizontal and vertical coordinates. This process allowed for the tracking of eye movements and the analysis of gaze vectors (Figure 4).

### Performance metrics for model validation

The mean Intersection over Union (mIoU) score, which represents the ratio of the predicted area overlap to the actual area overlap, was employed to assess the performance of the segmentation model. A value closer to 1 indicates better model performance in accurately segmenting the desired areas;


$$\begin{array}{c} J\left(GT,\;P\right)=1-\;\frac{GT\cap P\;}{GT\cup P}\\ \left(GT:ground\;truth,\;P\;predicted\;values\right) \end{array}$$

The mean absolute error (MAE), which calculates the average absolute difference between observations and estimates, and the root mean squared error (RMSE), which computes the average square of the difference, were utilized for model validation of eye tracking. Lower RMSE and MAE values, closer to 0, indicate superior model performance, implying that the observed and estimated values are closely aligned;


$$\begin{array}{c} MAE=\frac{1}{n}\overset{n}{\underset{i=1}{\sum }}|\hat{Y_{i}}-Y_{i}|,RMSE=\sqrt{\frac{1}{n}\overset{n}{\underset{i=1}{\sum }}{\left(\hat{Y_{i}}-Y_{i}\right)}^{2}} \end{array}$$

where *Y*_*i*_ is the *i*th observation, $\hat{Y_{i}}$ is the *i*th estimate, and *n* is the number of data points.

The study conducted a comparative analysis, evaluating the performance and inference times of the gaze-tracking and ocular object segmentation models in relation to findings from previously developed models.

## Results

### Model performance for ocular object segmentation

In our initial evaluation, we focused on assessing the performance of our ocular object segmentation model. For a detailed description of the algorithm, please refer to the Supplementary material. We conducted a comparative analysis with open-source models, ensuring that all input images were appropriately adjusted to meet the varying size requirements of different models (as outlined in Table 3).

**Table 3:** Segmentation model performance comparison using mean IoU scores.

**Model (W, H, C)**	**OpenEDS**			**HUSHH**		
	**Sclera**	**Iris**	**Pupil**	**Sclera**	**Iris**	**Pupil**
Deep VOG (240, 320, 3)	-	-	0.6806	-	-	0.8171
EllSeg (240, 320, 1)	-	0.8278	0.9157	-	0.6607	0.7632
RITnet (640, 400, 1)	0.0290	0.4663	0.7142	0.0674	0.4157	0.5404
**Our model (100, 100, 1)**	**0.9743**	**0.9601**	**0.9281**	**0.9500**	**0.9352**	**0.9073**

(W, H, C), optimal image size for each model. The model with the highest mean IoU scores, denoted in bold, indicates superior performance (higher scores represent better performance).

Interestingly, the DeepVOG model (14) exhibited higher accuracy in predicting images sourced from the HUSHH dataset. In contrast, EllSeg (15) and RITnet (16) demonstrated higher performance when dealing with OpenEDS images, reflecting their adaptability to the specific characteristics of the training data used for those models. However, when considering overall object segmentation performance across different data sources, our model consistently outperformed the alternatives. Irrespective of the data origin, our model consistently delivered superior results in terms of ocular object segmentation.

To assess pupil tracking performance (please refer to the Supplementary material for details on the algorithm), we computed metrics including, MAE, and RMSE. When validating with OpenEDS data, our algorithm achieved an average MAE of 0.42, and when using HUSHH data, the MAE was 0.59. In contrast, the GazeML eye-tracking model (17) yielded MAE values of 10.87 and 7.23 when applied to OpenEDS and HUSHH data, respectively. Notably, our model exhibited significantly superior performance compared to the GazeML model (Table 4).

**Table 4:** Eye-tracking performance comparison.

**Model**	**OpenEDS**		**HUSHH**	
	**MAE**	**RMSE**	**MAE**	**RMSE**
GazeML	10.8796	14.4496	7.2380	14.9750
**Our model**	**0.4220**	**0.5294**	**0.5953**	**0.8216**

The model with the lowest MAE and RMSE values, denoted in bold, indicates superior performance (lower values represent better performance).

The blink detection model (please refer to the Supplementary material for details on the algorithm) demonstrated high accuracy, achieving F1 scores of ≥ 0.95 for the open, closing, and closed states, along with area under the curve (AUC) values exceeding 0.99. Our model's predictions exhibited a high level of precision and reliability in blink detection (Table 5).

**Table 5:** Blink model performance.

**Label**	**AUC**	**F1**	**ACC**
Open	0.9954	0.9666	0.9786
Closing	0.9909	0.9502	0.9663
Closed	0.9985	0.9776	0.9877

### Execution speed and number of parameters

To ensure a fair comparison of execution speeds, we standardized all models by converting them to ONNX format and evaluating their performance consistently. We measured frames per second (FPS) values over 3,000 iterations with a batch size of 1 for this evaluation. The validation environment was set up on an Ubuntu 20.04 LTS operating system, AMD Ryzen 5 2600 and Intel Xeon Gold 5218R CPUs, as well as RTX3060 and RTX A6000 GPUs. The programming languages employed were Python (version 3.8.13) along with the ONNX Runtime-GPU library (version 1.14.1).

Remarkably, our model exhibited the swiftest ocular object segmentation performance. Despite EllSeg and RITnet having fewer parameters than our model, their runtimes were slower than ours across both the RTX3060 and RTX A6000 GPUs, as outlined in Table 6.

**Table 6:** Number of parameters and runtime of the ocular segmentation models.

**Model**	**Number of parameters**	**Frames per second**	
		**RTX 3060 (12.7 TFlops)**	**RTX A6000 (38.7 TFlops)**
DeepVOG	24,706,387	27.85	100.34
EllSeg	2,181,179	30.40	61.35
RITnet	248,900	53.70	116.32
**Our model**	**10,338,172**	**94.59**	**194.69**

TFlops, Tera Floating-point Operations Per Second. The model with the highest FPS, denoted in bold, indicates superior performance (higher values represent better performance).

### Nystagmus measurement

**Figure 5:**
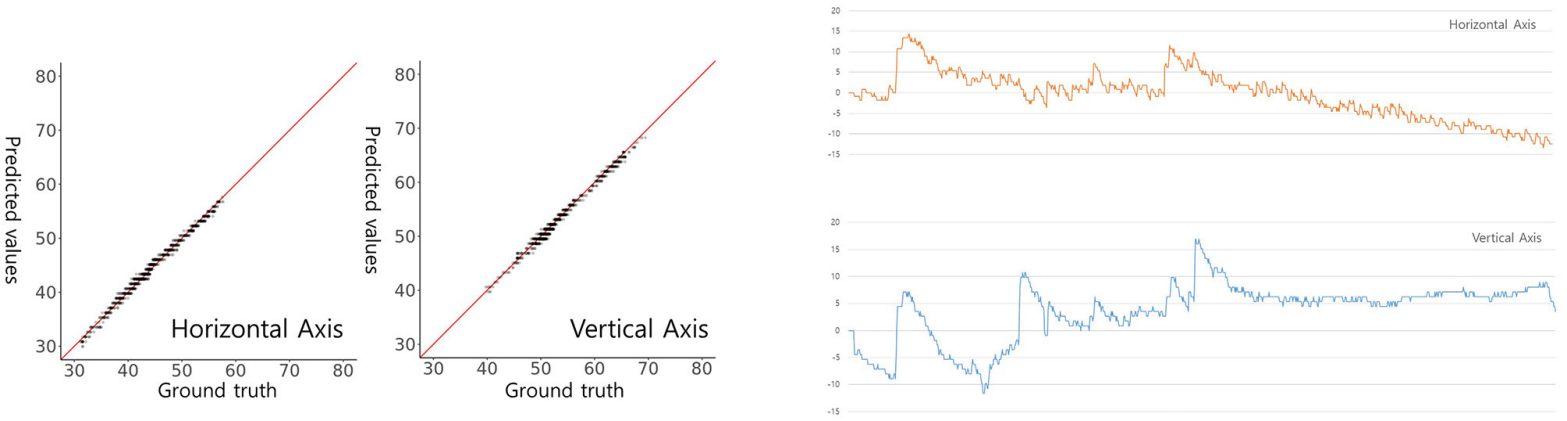
Model-predicted and actual pupil coordinates of a patient with BPPV **(left)** and a graphical presentation **(right)**. Scatterplots were drawn to assess the similarity between predicted and actual pupil coordinates. Each graph plots the predicted (y-axis) against the actual (x-axis) pupil coordinates. The red line indicates a perfect match (y = x). The horizontal (x-axis) and vertical (y-axis) centroid pupil coordinates inferred by our model on videos of BPPV patient eye movements are shown and evaluated on a per-frame basis.

Finally, we conducted an assessment of the performance of our trained model using image datasets to detect and classify horizontal and vertical nystagmus from eye movement videos. To achieve this, we randomly selected video clips recording the positional nystagmus of a patient with posterior canal BPPV in a dataset we had previously collected and curated (7). In this video clip, eye movements over time in the x (horizontal) and y (vertical) directions from the pupil center were computed by the ocular object segmentation model. When we employed the computed eye movements as the ground truth, representing the genuine reference data for nystagmus tracking. this reference data was compared with the predictions made by our model. We observed strong linear correlations between the ground truth eye movements and our model's predictions. Specifically, the correlation coefficient for horizontal eye movements was 0.9949, and that for vertical eye movements was 0.9950. Notably, both correlations demonstrated high statistical significance (Figure 5, *p* < 0.05), underscoring the robustness of our model's predictions. In summary, our model exhibited a potential possibility to detect and classify nystagmus, offering promising prospects for applications in clinical diagnosis and monitoring.

## Discussion

In cases of acute vertigo, a significant proportion of patients initially seek medical attention at EDs or PHCUs. It is worth noting that careful observations of eye movements can play a critical role in differentiating posterior circulation stroke, surpassing the sensitivity of neuroimaging modalities (1–4). However, in many of these clinical settings, routine evaluations of eye movements are not standard practice because front-end physicians often lack familiarity with these specialized tests and may feel uncertain about accurately interpreting the results (4). Additionally, a challenge arises from the fact that patients experiencing vertigo may not always seek medical attention at clinics when symptoms are actively occurring.

Even though telemedicine concepts using HINTS evaluation have been introduced for those scenarios (18, 19), it is important to acknowledge that implementing these concepts comes with limitations, particularly in terms of the additional resources required. This includes the need for teleconsultants, specialized equipment for detecting eye movements, and the establishment of robust network systems. Our earlier deep learning model, which classified nystagmus and localized BPPV, demonstrated high sensitivity and specificity with reasonable diagnostic performance (7). However, the model was trained using the dataset of positional tests conducted by experts, and the machine learning required a high-end GPU and considerable computing time. Thus, we thought that our earlier model also may not be practical in these clinical scenarios.

In light of these considerations, our primary objective has been to simplify and enhance the process of conducting eye movement examinations and interpreting them in these clinical scenarios. As a first step, our novel AI-based algorithm was designed to track eye movements or nystagmus in eye videos, with future improvements planned to enable compatibility with recordings from a webcam or smartphone camera. To achieve this, we have developed three AI models, including the ocular object segmentation model, blink detection model, and pupil center tracking model, which we subsequently integrated. This multi-faceted approach has allowed us to accurately identify and monitor nystagmus, providing valuable diagnostic information from the eye video itself.

For instance, our model initiates by detecting ocular elements, including the sclera, iris, and pupil, within eye video footage. Subsequently, it tracks eye movement by identifying pupil center coordinates and monitoring blinking over time, allowing us to measure and analyze eye movement directly from video footage.

In terms of ocular object segmentation, our model achieved outstanding mIoU scores for different eye components. Specifically, when we validated our model using the OpenEDS dataset, it achieved mIoU scores of 0.974 for the sclera, 0.9601 for the iris, and 0.9281 for the pupil. When we utilized the HUSHH dataset for validation, the scores remained impressive at 0.9500 for the sclera, 0.9352 for the iris, and 0.9073 for the pupil. These results surpassed the performance of other open-source models, as summarized in Table 3.

When validated using the OpenEDS dataset for eye-tracking performance, it achieved an MAE of 0.42. Similarly, when tested on the HUSHH dataset, it achieved an MAE of 0.59. In contrast, the GazeML model (17), when validated on the same datasets, yielded much higher MAEs of 10.87 for OpenEDS and 7.23 for HUSHH. These results clearly highlight the superior accuracy and precision of our model in the context of eye-tracking.

However, it's important to acknowledge that, despite excluding frames with similar features from our dataset using a similarity index during model development, our model has not yet been validated with a truly independent dataset. This highlights the necessity for additional validation using an independent dataset to fully establish the model's efficacy and generalizability.

Interestingly, despite its initial training involving virtual eye images—synthetic images generated by UnityEyes within a virtual reality (VR) environment—our model has demonstrated remarkable performance when applied to real-world footage In fact, synthetic data has played a crucial role in augmenting datasets, particularly in scenarios where generating data within a clinical setting is difficult. This type of data enables the training of AI models using realistic simulations, which is especially beneficial when clinical data is scarce. Therefore, we speculated that employing synthetic data in model training would lead to improved accuracy in the final AI model for nystagmus tracking.

While it is well-known that open-source models, as outlined in Table 3, provide segmentation functions by classifying ocular elements, our model stood out as particularly well-suited for eye tracking compared to other models for several reasons. Firstly, the current DeepVOG does not provide predictions for the sclera and iris (14). Similarly, EllSeg does not offer predictions for the sclera (15). Even though RITnet provides predictions for the sclera, iris, and pupil (16), it exhibits very low IoU performance on benchmark datasets. Moreover, apart from our model, all other models showed a discrepancy of 0.10 or more in IoU between OpenEDS and HUSHH, indicating poor generalization performance. Thus, it is evident that DeepVOG, EllSeg, and RITnet were not suitable models for achieving accurate segmentation for nystagmus measurement, even when used as backbones. However, to enhance the robustness of our model's performance, it is crucial to incorporate alternative validation strategies, including the use of additional datasets.

Subsequently, we evaluated the potential performance of our model. We conducted experiments with the ocular object segmentation model that we employed in two different systems: a consumer-grade system equipped with an RTX 3060 GPU and an AMD Ryzen 5 2600 CPU, and a workstation-grade system running Ubuntu 20.04 LTS with the RTX A6000 GPU and Intel Xeon Gold 5218R CPUs. The achieved frame rates were 94.59 FPS for the former and 194.69 FPS for the latter. These results significantly surpass the performance of existing models. Although EllSeg (15) and RITnet (16) have fewer parameters than our model during ocular segmentation tasks, their runtimes were slower. These results suggest that our model could be well-suited for potential deployment on low-end computers. Its efficiency and speed make it a promising choice for such resource-constrained platforms.

In our final evaluation for nystagmus measurement, we adopted a manual approach to determine the pupil center's coordinates on both the x (horizontal) and y (vertical) axes. These coordinates were extracted from images using our ocular object segmentation model, derived from video frames of a patient with posterior canal BPPV. Subsequently, changes in these coordinates over time were computed and used as the ground truth data, representing the actual eye movements. The results were highly promising, with the correlations between our model's predictions and the ground truth data demonstrating excellent agreement. Specifically, the correlations were 0.9949 for horizontal movements and 0.9950 for vertical movements. Our model showed its capability to accurately classify both the horizontal and vertical components of nystagmus within eye videos.

However, it is important to acknowledge the limited scope of our dataset, which consists of video analysis from a single BPPV patient. Despite generating 1,109 segmentation masks from this patient's video footage within the BPPV datasets, our approach should primarily be viewed as a proof-of-concept. It demonstrates the model's capabilities in a specific and well-defined condition. For future clinical applications, this model should be validated with a larger dataset to ensure its efficacy and reliability in a clinical context.

We should address that nystagmus or nystagmus-like eye movements are typically best evaluated using infrared goggles with an eye tracker or Frenzel's glasses to diminish visual fixation. However, tests like the HI test, gaze-evoked nystagmus observations, and TS are usually conducted in well-lit spaces. Additionally, spontaneous nystagmus following acute vestibular function loss or abnormal eye movements due to central lesions can be observed in situations where there is no precise control over visual surroundings. In this light, our model's ability to track nystagmus from eye videos has significant clinical implications for remote diagnosis and home-based monitoring, eliminating the need for additional equipment such as an eye tracker.

Recent advancements have introduced mobile-centric models for nystagmus tracking, such as ConVNG (9) and EyePhone (20), which are designed for smartphones. Our model, however, is not merely a standalone tool for nystagmus detection but rather a foundational model making significant contributions to this field. While it shares similarities with models like ConVNG, key distinctions are in the methods of model inference, the variety of training datasets utilized, and the methods employed for validation. Firstly, our model was trained using segmentation masks for the iris, pupil, and sclera, specifically for nystagmus tracking. In contrast, ConVNG tracks 17 different landmarks in the ocular region. Secondly, our model's training incorporated real-world data from various vertigo patients and synthetic datasets, whereas ConVNG was trained with optokinetic stimuli. In terms of validation, ConVNG has been tested with two independent clinical datasets covering a range of eye movement disorders, whereas our validation focused on footage from BPPV patient. While recognizing ConVNG (9) and EyePhone (20) as a notable contribution in the field of neurotology, our model with its simpler design also demonstrated reasonable performance, indicating its potential for accurate nystagmus classification in clinically relevant scenarios. However, further validation with diverse datasets is necessary to fully establish its effectiveness.

In summary, our algorithm showed superior capabilities in ocular object segmentation and eye-tracking when compared to conventional algorithms. It excels in providing a high FPS rate and can perform edge computing to real-time analysis with high accuracy. Currently, the model effectively tracks horizontal and vertical eye movements from video clips. Future development efforts will focus on expanding the model's capabilities to detect torsional eye movements and incorporating diagnostic support functions derived from structured neuro-otological examinations. This advancement holds the potential to provide patient-centered solutions by enabling the detection and recording of eye movements. However, before widespread deployment, it is essential to conduct an international multi-institutional study for external cross-validation, adhering to the CONSORT-AI extension guidelines (21). This rigorous validation process will ensure the reliability and generalizability of our model's performance across different clinical settings and populations, ultimately enhancing its utility in healthcare applications.

## Data availability statement

The original contributions presented in the study are included in the article/Supplementary material, further inquiries can be directed to the corresponding authors.

## Ethics statement

The studies involving humans were approved by the Institutional Review Board of Hallym University College of Medicine approved our use of the datasets (#2022-02-012). The studies were conducted in accordance with the local legislation and institutional requirements. Written informed consent for participation was not required from the participants or the participants' legal guardians/next of kin in accordance with the national legislation and institutional requirements.

## Author contributions

CC: Conceptualization, Data curation, Formal analysis, Methodology, Validation, Writing – original draft. SP: Data curation, Formal analysis, Software, Validation, Writing – original draft. SM: Formal analysis, Methodology, Writing – original draft. H-JL: Data curation, Formal analysis, Writing – review & editing. E-CL: Conceptualization, Data curation, Formal analysis, Methodology, Writing – original draft, Writing – review & editing. SH: Conceptualization, Formal analysis, Funding acquisition, Supervision, Writing – review & editing.
