# Supplementary material for: Feasibility of video-based real-time nystagmus tracking: a lightweight deep learning model approach using ocular object segmentation
Source: Front Neurol. 2024 Feb 21;15:1342108. doi: 10.3389/fneur.2024.1342108 (PMC10915048; doi:10.3389/fneur.2024.1342108)
Supplement: Supplementary file 1 [file Data_Sheet_1.DOCX]

1. *Ocular object segmentation model*

Our model for ocular object segmentation used the Efficient (B0) model developed by Tan et al. as a Backbone for real-time inference (Tan and Le, 2019). EfficientNet proposes a way to improve accuracy while reducing model size and computation by finding the optimal combination of depth, width, and resolution that directly impact performance under limited resources. Increasing the network depth increases the model capacity, allowing for more complex features to be captured, but it also makes training more difficult due to the vanishing gradient problem. Several techniques, such as batch normalization and residual connections, have emerged to address this issue. Adjusting the width and resolution of each layer improves accuracy, but it increases computation proportional to the square. The process of finding the optimal combination is called compound scaling, and it resulted in a model that is 8.4 times smaller and 6.1 times faster than the existing ConvNet, while not significantly sacrificing accuracy. EfficientNet offers models with different parameter sizes ranging from B0 to B7. B0 has the smallest number of parameters at 5.3 million, while B7 has the largest with 66 million parameters. In this paper, The training process utilized Adam optimizer with a batch size of 32 for 50 epochs and a learning rate of 0.05. Two schedulers were also implemented, including ReduceLROnPlateau, which adjusts the learning rate if no improvement is observed, and Early Stopping, which terminates learning early if no progress is made. For ReduceLROnPlateau, the learning rate was reduced by a factor of 0.2 with a patience value of 2, while Early Stopping used a patience of 10 to halt training when no improvement was observed. The sum of Binary Cross Entropy and Jaccard Loss was used as the loss function for the training process.

$BCELoss\left( GT, P \right)= -[GT\cdot\log\left( P \right)-\left( 1-GT \right)\cdot\log\left( 1-P \right)]$, $J\left( GT, P \right)=1- \frac{GT\cap P}{GT\cup P}$

$$GT :Ground Truth, P :Prediction Values)$$

1. *Blink detection model*

The blink model was also constructed by referencing the model structure of EfficientPose (Groos et al., 2021) for real-time inference. EfficientPose has improved the disadvantage of the existing posture estimation algorithm, which cannot be used in real-time due to its high computational load (Groos et al., 2021). The EfficientPose network uses a Detection Block to train a heatmap, which is structured to efficiently use memory space. In this study, the DetectionBlock was combined with the Block3B output of the previously developed ocular object segmentation model to generate a classification model. The batch size was set to 32, the epoch was set to 30, and the learning rate was set to 0.01, with RMSProp used as the optimizer. ReduceLROnPlateau and Early Stopping schedulers were used, with a factor of 0.2 and patience of 2 set for ReduceLROnPlateau, and a patience of 10 set for Early Stopping. Cross Entropy was used as the loss function.

1. *Eye Tracking Model*

The eye tracking model, like the blink model, also used the Block3B output and the Detection Block. The eye tracking model was configured as a model structure with a HeatMap of size of 112x112 and a center coordinate value of pupil as target by performing up-scaling at 112x112 after the Detection Block. The batch size was set to 64, the epoch to 50, the learning rate to 0.001, and Adam was used as the optimizer. We used ReduceLROnPlateau and Early Stop scheduler as schedulers. The factor of ReduceLROnPlateau was set to 0.2 and the patience was set to 2. The patience of Early Stopping was set to 10. In addition, the loss function was Cross Entropy and Mean Squared Error (MSE)

Groos, D., Ramampiaro, H., and Ihlen, E.A. (2021). EfficientPose: Scalable single-person pose estimation. *Applied intelligence* 51**,** 2518-2533.

Tan, M., and Le, Q. (Year). "Efficientnet: Rethinking model scaling for convolutional neural networks", in: *International conference on machine learning*: PMLR), 6105-6114.
